# ASO Author Reflections: Addressing Hospital-Based Disparities in Access to Cytoreductive Surgery with Hyperthermic Intraperitoneal Chemotherapy (CRS-HIPEC) and Survival in Patients with Isolated Colorectal Peritoneal Metastases

**DOI:** 10.1245/s10434-024-15193-0

**Published:** 2024-03-18

**Authors:** Roos G. F. M. van der Ven, Teun B. M. van den Heuvel, Koen P. B. Rovers, Simon W. Nienhuijs, Felice N. van Erning, Ignace H. J. T. de Hingh

**Affiliations:** 1 Department of Research & Development, Netherlands Comprehensive Cancer Organisation (IKNL), Utrecht, The Netherlands; 2 Department of Health Services Research, Faculty of Health, Care and Public Health Research Institute (CAPHRI), Faculty of Health, Medicine and Life Sciences, Maastricht University, Maastricht, The Netherlands; 3 Department of Oncology and Developmental Biology (GROW), Faculty of Health, Medicine and Life Sciences, Maastricht University, Maastricht, The Netherlands; 4 Department of Surgery, Catharina Hospital, Eindhoven, The Netherlands

## Past

Colorectal cancer (CRC) ranks among the most commonly diagnosed cancers worldwide.^1^ More than 5% of patients with CRC present with synchronous peritoneal metastases (PMCRC).^2^ In the Netherlands, select patients with isolated and limited PMCRC are treated with curative intent cytoreductive surgery (CRS) combined with hyperthermic intraperitoneal chemotherapy (HIPEC).^3^ This treatment is exclusively performed at specialized HIPEC centers, while isolated PMCRC are diagnosed in all Dutch hospitals. However, a previous study showed that between 2009 and 2015 significant disparities in the odds of receiving CRS-HIPEC, and subsequently survival, existed on the basis of the hospital of diagnosis being either a specialized HIPEC center or a referring hospital.^4^ This resulted in patients possibly missing out on curative or life-prolonging treatment. To reduce this unintended variation between hospitals of diagnosis, national efforts were launched, including education on PMCRC, the inclusion of CRS-HIPEC in guidelines, the establishment of a national multidisciplinary working group (the Dutch Peritoneal Oncology Group, DPOG), initiation of nationwide prospective studies, and the enhancement of referral networks.

## Present

Using real-world data, the present nationwide observational cohort study in patients with isolated synchronous PMCRC assessed the association between hospital of diagnosis (HIPEC center versus referring hospital) and the probability of undergoing CRS-HIPEC, and subsequent survival in the periods before (2009–2015) and after (2016–2021) aforementioned national efforts were initiated. Overall, the percentage of patients undergoing CRS-HIPEC increased from 17.2% in the first period (2009–2015) to 23.4% in the latter period (2016–2021). In the first period, patients diagnosed in HIPEC centers had a significantly higher odds of undergoing CRS-HIPEC [adjusted odds ratio (OR) 1.64, 95% confidence interval (CI) 1.02–2.67] and better survival [adjusted hazard ratio (HR) 0.80, 95% CI 0.66–0.96] compared with patients from referring centers. However, in the latter period, the differences in odds of undergoing CRS-HIPEC (OR 1.27, 95% CI 0.76–2.13) and survival (HR 0.80, 95% CI 0.66–0.96) were no longer statistically significant.^5^ These data suggest that national efforts initiated to reduce unintended interhospital variation may thus have contributed to equal access to care and a similar chance of survival for patients diagnosed with isolated synchronous colorectal PMCRC at a national level.

## Future

The likelihood of receiving a specific treatment should not depend on the hospital of diagnosis, as this may result in patients being deprived of potentially curative or life-prolonging treatments. The results of the present study do not only underscore the importance of observational population-based data to recognize and rectify treatment and survival discrepancies between hospitals within a region or country, but also show that national efforts can make a difference in reducing unwarranted inter-hospital variation in access to care. These findings could provide an example for other countries and fields aiming to minimize disparities in medical practices between hospitals on a broader scale, thereby potentially improving access to optimal treatment and chance of survival for every patient.
